# Not all players warm up the same: position-specific external load during standardized pre-match protocol in women’s handball

**DOI:** 10.3389/fspor.2026.1881325

**Published:** 2026-08-18

**Authors:** Carlos García-Sánchez, Álvaro Caro Serrano, Reidel Cordoves Peinado, Raúl Nieto-Acevedo, Moisés Marquina Nieto, Alfonso de la Rubia

**Affiliations:** 1 Deporte y Entrenamiento Research Group, Departamento de Deportes, Facultad de Ciencias de la Actividad Física y del Deporte (INEF), Universidad Politécnica de Madrid, Madrid, Spain; 2 Universidad Pedagógica Nacional Francisco Morazán, Universidad Nacional Autónoma de Honduras, Tegucigalpa, Honduras; 3 Facultad de Ciencias Biomédicas y de la Salud, Universidad Alfonso X el Sabio (UAX), Madrid, Spain; 4 Campus de Ciencias de la Vida, Universidad de Nebrija, Madrid, Spain

**Keywords:** inertial measurement units, local positioning system, player monitoring, team sport, tracking system, ultra-wideband technology, wearable device

## Abstract

**Introduction:**

Although monitoring external load during training and match play is essential to understand game demands, optimize performance, and reduce injury risk, the external load accumulated during the pre-match warm-up has received little attention in women's handball. Therefore, the aims of the present study were: (i) to analyze and compare positional differences in the external load registered during a standardized pre-match warm-up protocol in official matches in women's handball; and (ii) to examine the external load accumulated during the warm-up relative to match demands according to playing position.

**Methods:**

Twenty-two female players from the Spanish 2nd Division were monitored across 13 official home matches and their respective pre-match warm-ups. Total distance covered (TDC), high-speed running (HSR), metabolic power (MP), high metabolic load distance (HMLD), high-intensity accelerations (HIA), high-intensity decelerations (HID), and PlayerLoad (PL) were recorded in absolute and relative values (normalized by playing time) using a WIMU PRO™ LPS integrating UWB and IMU technology. Positional differences were analyzed using one-way ANOVA or Kruskal–Wallis test. The statistical significance level was set at *p* < 0.05.

**Results:**

Significant between-position differences were found in TDC, TDC/min, HSR, HSR/min, HMLD, HMLD/min, MP/min, PL, and PL/min (*p* < 0.05). Overall, wings exhibited the greatest external load values during the warm-up, particularly in TDC, HSR, HMLD, and PL, whereas pivots consistently showed the lowest values. Backs also displayed higher TDC/min, HMLD/min, and MP/min than pivots. When warm-up load was contextualized relative to match demands, the warm-up accounted for a considerable proportion of match load in several variables. Pivots generally showed the greatest relative contribution of warm-up to match load, whereas wings showed the lowest values across most variables.

**Conclusions:**

External load registered during a standardized pre-match warm-up in women's handball is position-specific and should not be considered a uniform preparatory stimulus across players. Warm-up load represents a meaningful component of total match-day external load and should be structured according to the technical and physical demands of each playing position. These findings support the individualization of pre-match warm-up strategies according to playing position.

## 1 Introduction

Handball is a team sport characterized by its intermittent and unpredictable and chaotic nature, alternating high-intensity actions with variable low-intensity recovery periods (1). During a match, players perform repeated running, sprinting, acceleration, deceleration, change-of-direction, and jumping actions, frequently combined with high-demanding physical contact against opponents (2, 3). These actions impose substantial neuromuscular, metabolic, and mechanical demands on players (4, 5).

Consequently, understanding the physical demands of handball has become a priority in both research and applied practice (6). In this context, the need to quantify physical demands with a good level of validity (7), and reliability (8) has driven significant advancements in monitoring technologies applied to indoor sports such as handball (9). Over the past decade, advances in hardware and processing have enabled local positioning technology (LPS), using ultra-wideband (UWB) systems and inertial measurement units (IMU) (e.g., accelerometer, magnetometer and gyroscope), to provide more reliable and sensitive results in the real-time measurement of kinetic, metabolic, and mechanical variables (10). This evolution has represented a substantial shift from traditional methods (i.e., time-motion analysis) (11), enabling a more detailed description of variables such as distance, speed, linear acceleration, or mechanical load, and helping to guide key processes such as training prescription, return-to-play assessment, or injury risk management (1).

Nevertheless, external load values in handball are influenced by multiple factors, including competitive level, sex, match phase, and, especially, playing position (6, 12–15). The total distance covered was largely greater in national competitions (4,506.7 ± 647.9 m) compared with international competitions (2,190.3 ± 1,950.5 m), while women have been shown to cover more meters per minute than men (110.5 ± 7.2 vs. 78.4 ± 19.7 m·min^−1^, respectively) (6). Match demands also decline in some high-intensity metrics toward the final stages of the game, with high-speed running decreasing from 44.5 ± 37.8 m during the first 5 min of the match to 16.3 ± 8.1 m during the final 5 min phase (12). Among these factors, playing position appears to be one of the clearest determinants of external load. Backs and wings cover greater total distances (3,903 ± 1,224 m and 3,571 ± 864 m, respectively) and slightly more meters per minute (64.5 ± 10.4 m·min^−1^ and 61.8 ± 7.8 m·min^−1^, respectively) than pivots (3,149 ± 630 m and 56.5 ± 6.6 m·min^−1^, respectively) (13). In contrast, center backs accumulate more high-intensity decelerations than pivots (142.7 ± 59.5 vs. 99.6 ± 28.9) (13). Additionally, a recent study conducted in EHF EURO 2024, showed that wings, and line players perform a higher number of high-intensity actions than line players (60, 58, and 43 actions per match, respectively) (14). In summary, the current scientific evidence showed that wings register the greatest locomotor demands, whereas pivots display the lowest movement demands but a high frequency of impacts, reflecting their frequent involvement in physical duels and positional play in restricted spaces (15).

Athlete monitoring in handball has mainly focused on training sessions and competitive match play to describe physical demands and support load-management decisions (6, 9). However, the pre-match warm-up also contributes to the external load accumulated on match day. Despite this, it is rarely considered as a distinct monitoring period. Therefore, excluding the warm-up may underestimate the total competition-day load experienced by players. Likewise, although competitive load has been extensively described in relation to playing position, there is still a lack of evidence regarding whether these positional differences are also present during the pre-match warm-up in handball. Technical staffs design and implement these protocols to progressively prepare athletes physically and psychologically for competition (16). Warm-up routines increase body temperature, promote neuromuscular activation, and enhance acute readiness for performance (17, 18).

Standardized warm-ups are commonly applied to the entire team, even though players perform different positional roles. Consequently, an identical protocol may produce unequal external load responses and may not prepare every player equally for subsequent match demands. Evidence from other team sports, such as soccer and volleyball, showed significant between-position differences in variables such as distance covered, jumps, accelerations, and mechanical load, depending on the player's specific role and competitive context (19, 20). This highlights the need to determine whether a standardized warm-up adequately reflects the specific demands of each playing position or, conversely, contributes to unequal preparatory loads before competition. Therefore, the aims of the present study were: (i) to analyze and compare the differences between playing positions in the external load registered during a standardized pre-match warm-up protocol in official matches in women's handball; and (ii) to examine the external load accumulated during the warm-up relative to match demands according to playing position. To the best of the authors’ knowledge, this is the first study to examine position-specific external-load demands during a standardized pre-match warm-up in women's handball. Based on a similar study conducted in elite female volleyball players (19), it was hypothesized that external load would differ depending on playing positions, with wings and backs accumulating greater locomotor load than pivots.

## 2 Material and methods

### 2.1 Participants

Twenty-two semi-professional female handball players participated in the study. Playing positions were: wings (*n* = 4; age: 18.8 ± 0.5 years; height: 162.0 ± 3.8 cm; body mass: 55.5 ± 4.3 kg), backs (*n* = 14; age: 20.9 ± 3.6 years; height: 168.7 ± 3.9 cm; body mass: 65.4 ± 6.8 kg) and pivots (*n* = 4; age: 21.0 ± 1.8 years; height: 171.3 ± 4.8 cm; body mass: 79.1 ± 11.0 kg). The investigated team was the club's first women's team rather than a reserve or development squad. According to the Participant Classification Framework proposed by McKay et al. (21), the players were classified as highly trained or national-level athletes (Tier 3). During the season, players typically completed four or five handball training sessions, two or three strength training sessions and one match per week. All players were informed of the study requirements and provided written informed consent prior to the start of the study. Additionally, all the ethical procedures used in this study were in accordance with the Declaration of Helsinki and were approved by the Ethics Committee of the European University of Madrid (CIPI/ 18/195).

### 2.2 Design

An observational longitudinal study was conducted to analyze and compare the differences between playing positions on external load encountered by female handball players on a standardized pre-match warm-up. The reported results corresponded to the average values of 13 official home matches played and their respective pre-match warm-ups over 32 weeks during an 8-month (September to April) competitive season in the 2021–2022 Spanish 2nd National Division. In line with previous studies, goalkeepers were excluded from the analysis because running-based external load metrics do not reflect their performance needs (12–15, 23, 24). Players who were injured during the warm-up or the match were excluded from the analysis, resulting in a total of 153 individual LPS registers (wings, *n* = 39; backs, *n* = 88; and pivots, *n* = 26).

### 2.3 Procedures and data analysis

#### 2.3.1 Standardized warm-up protocol

**Figure 1:**
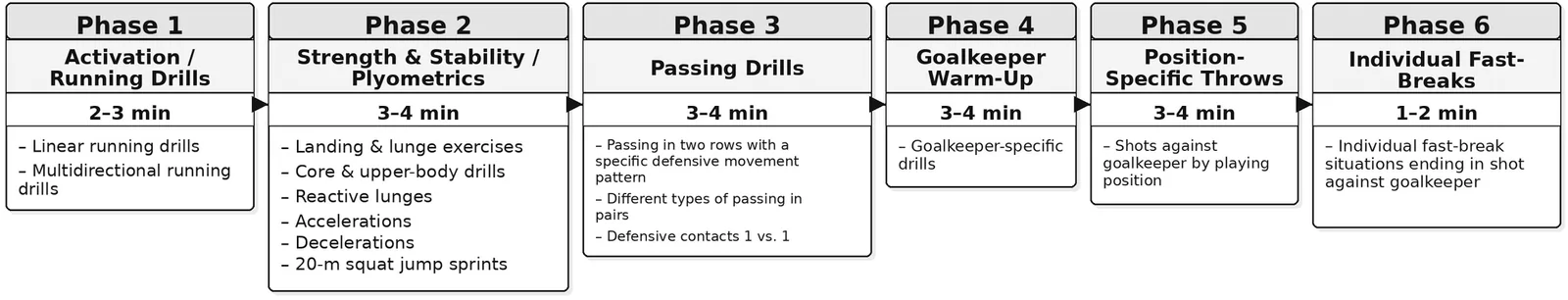
Standardized pre-match warm-up protocol performed before official matches.

Figure 1 illustrates the standardized pre-match warm-up protocol performed before all official matches. The protocol lasted ∼20 min and consisted of six consecutive phases, progressing from general physical preparation without the ball to handball-specific exercises.

The initial phase consisted of frontal running, lateral movements, and short running sequences with changes of direction. Phase 2 focused on lower-limb strength, bilateral and unilateral landing exercises, posterior-chain activation, and deceleration mechanics. The phase also included unilateral isometric and dynamic bridge exercises, frontal and lateral lunges, shoulder and upper-limb strength exercises, and trunk-stability tasks. These activities were combined with short accelerations, controlled decelerations, and changes of direction. The phase concluded with jump squats followed by 20-m sprints to provide plyometric and neural activation. In Phase 3, players subsequently worked in pairs facing each other and completed progressive passing sequences. At the end of these sequences, each pair performed three to four controlled one-on-one defensive stopping actions. During each action, the defender placed one arm against the partner's throwing arm and the opposite arm around the upper back or trunk, simulating the body-contact technique commonly used to control the attacker and interrupt the throwing action or forward progression. The following phase was devoted to goalkeeper preparation. Court players formed a single line in front of the goal and alternately performed standardized shots against each of the two goalkeepers. The shooting sequence progressed through predefined target areas: shots close to the goalkeeper's body, shots directed towards the upper corners, shots towards the lower corners, and shots at mid-height. Players then completed position-specific throwing actions and individual fast-break sequences before finishing with brief high-intensity handball-specific actions.

The same task order and general timing were maintained across all matches. During all warm-ups, the duration of each phase and the total protocol duration were independently controlled by both a member of the research team and the team's strength and conditioning coach, providing dual control of protocol timing. Statistical analysis confirmed that warm-up duration did not differ significantly between playing positions or between match days (*p* > 0.05). Additionally, before the start of the standardized team warm-up, some players voluntarily performed brief individual mobility exercises. However, these activities were neither prescribed nor systematically quantified and were therefore not included in the external-load analysis. Accordingly, the reported values refer exclusively to the standardized team warm-up.

#### 2.3.2 LPS system installation and calibration

All players were monitored using a local positioning system with ultra-wideband technology and inertial measurement units (WIMU PRO™, Hudl® SL, Nebraska, USA). Each device has a number and is affiliated with the same player for the entire season to limit problems that may exist due to inter-unit reliability of the devices (22). Players were already familiar with the monitoring procedures through their regular use during training sessions and friendly matches.

Prior to the match, the LPS system was installed on the official home handball court in accordance with the manufacturer's manual and guidelines described in previous studies (12, 13, 23, 24). Six UWB antennas were positioned around the court, forming a hexagonal configuration to optimize signal transmission and reception. Four antennas were placed approximately 5 m from the court perimeter lines, whereas the two antennas located adjacent to the center line were positioned approximately 7 m from the court perimeter lines. Once installed, the antennas were switched on sequentially, with the master antenna activated last. A 5-min automatic calibration process was then performed, during which the master antenna synchronized all antennas using a common clock.

Approximately 10–20 min before the start of the warm-up, the units were placed in the centre of the court, switched on, and allowed to establish automatic recognition and communication with the antenna network. Finally, a researcher walked along the court perimeter lines while carrying one unit to define the playing-area perimeter within the manufacturer's software. Each player then wore her assigned device between the shoulder blades in an adjustable vest.

Only the standardized team warm-up period was included in the analyses. Its start and end were identified using timestamps recorded by the researcher supervising the protocol, and any activity recorded before or after this period was excluded. Data were recorded in real time and subsequently processed using the manufacturer's software (SPRO™, version 2.3.0, Hudl® SL, Nebraska, USA). After each match, the files were downloaded, reviewed, exported in Excel format, and imported into the statistical software for analysis.

### 2.4 External load metrics

Based on previous publications examining the physical demands of handball (12, 13, 23, 24), seven external load metrics were collected in absolute and relative values (normalized by playing time) from the LPS and the IMUs. A detailed description of each external load metric monitored is provided in Table 1.

**Table 1:** Description of external load metrics.

Name	Unit	Description
Total distance covered (TDC)	Meters	Was defined as the total distance covered by the player
High speed running (HSR)	Meters	Was defined as the total distance covered above 18.1 km/h
Metabolic Power (MP)	W·kg^−1^	Was calculated as the product of the energy expenditure per unit of time necessary to move at a certain speed (i.e., running at a constant velocity of 5.5 m·s^−1^ or 19.8 km·h^−1^) and acceleration (i.e., accelerating from 2 to 4 m·s^−2^ for 1 s (25)
High metabolic load distance (HMLD)	Meters	Corresponded to the distance covered with a metabolic power consumption above 25.5 W·kg^−1^
High-intensity accelerations (HIA)	Count	Total number of accelerations above 3 m·s^−2^ performed by the player
High-intensity decelerations (HID)	Count	Total number of decelerations above 3 m·s^−2^ performed by player
PlayerLoad (PL)	a.u.	Was calculated as a vector magnitude expressed as the square root of the sum of the squared instantaneous rates of change in acceleration in each one of the three planes divided by 100 (26)

### 2.5 Statistical analysis

Descriptive statistics were presented as means and standard deviations (M ± SD). The Kolmogorov–Smirnov test was performed to confirm data distribution normality and Levene's test for equality of variances. Playing positions differences were determined by variance analysis one-way ANOVA followed by Tukey *post hoc* testing (parametric variables), or Kruskal–Wallis followed by the Dwass-Steel-Critch-low-Fligner test (non-parametric variables). Furthermore, partial eta-squared (*η_p_*^2^) or epsilon-squared (*ε*^2^) was calculated for group effects with the following interpretation: >0.01 small, >0.06 moderate, and >0.14 large (27). Cohen's effect sizes (ES) were calculated and interpreted using Hopkins’ categorization criteria: *d* > 0.2 as small, *d* > 0.6 as moderate *d* > 1.2 as large, and *d* > 2.0 as very large (28). The statistical significance level was set at *p* < 0.05. All statistical analyses were performed using the software JAMOVI (version 2.3.21.0, 2022) for Macintosh.

## 3 Results

**Figure 2:**
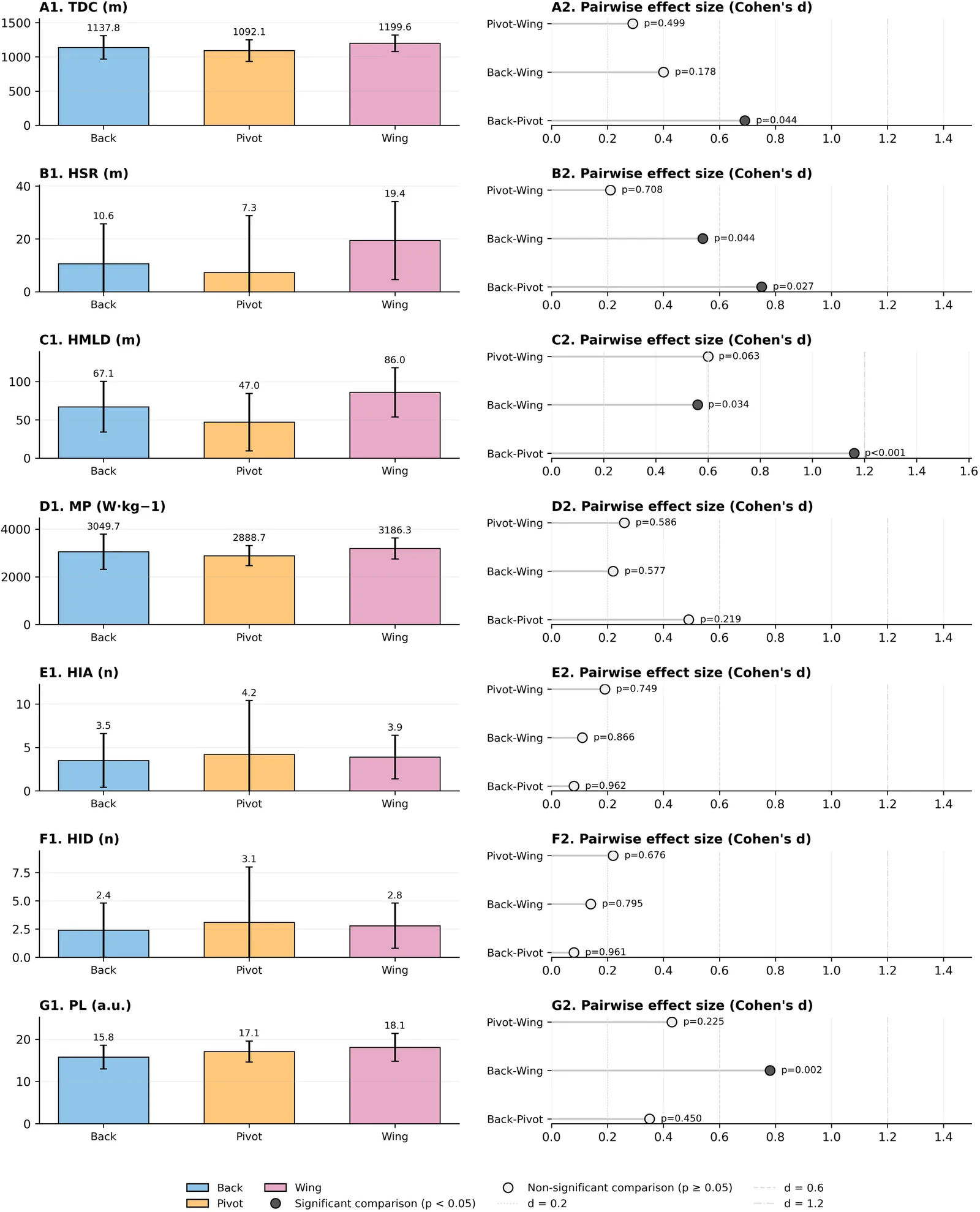
Absolute external load differences between playing positions during the standardized pre-match warm-up in women's handball. Panel **A** shows mean ± SD values by position, and Panel **B** shows pairwise comparisons with *p*-values and effect sizes.

**Figure 3:**
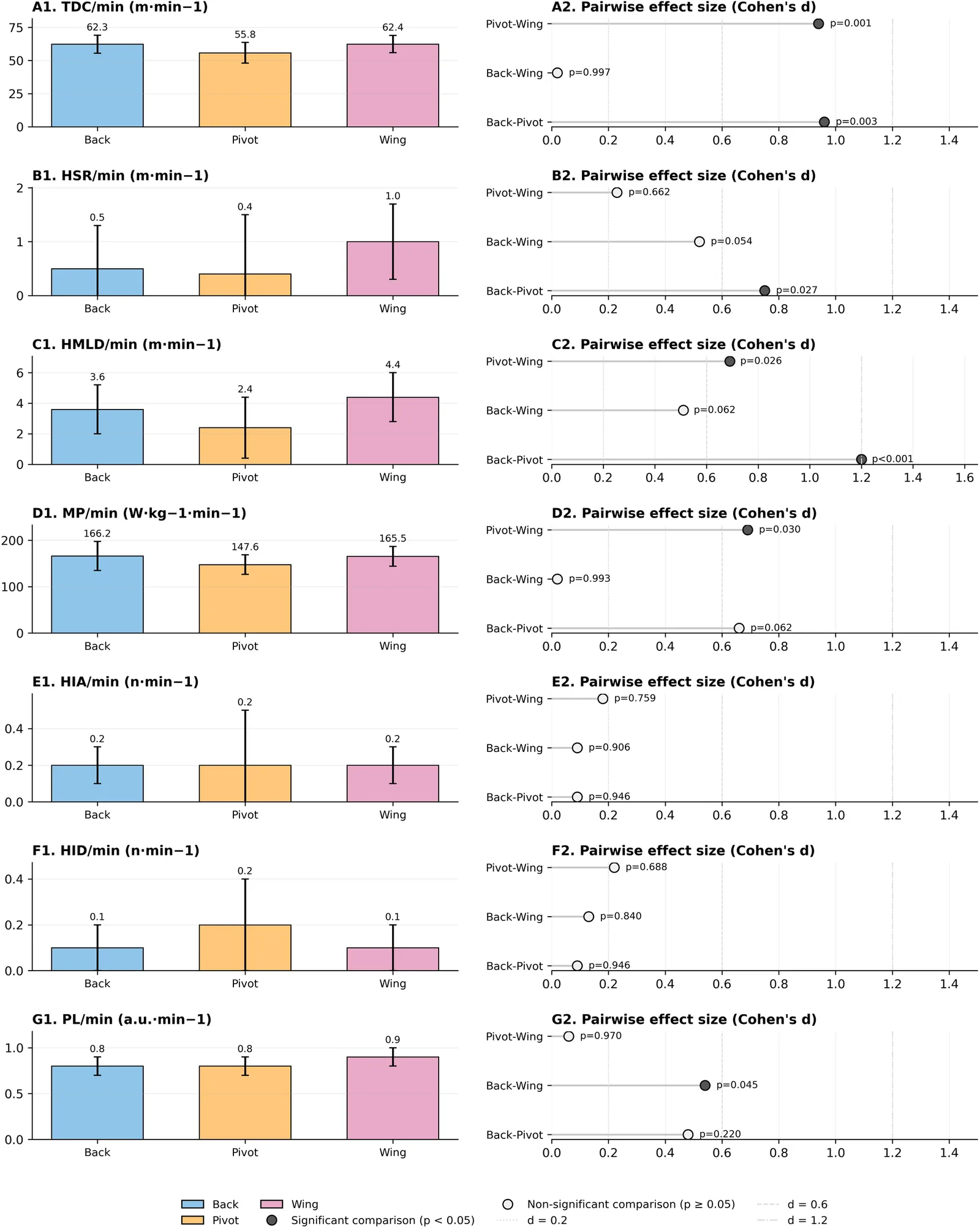
Relative external load differences between playing positions during the standardized pre-match warm-up in women's handball. Panel **A** shows mean ± SD values by position, and Panel **B** shows pairwise comparisons with *p*-values and effect sizes.

Descriptive and inferential results for absolute and relative external-load variables were presented in Table 2, whereas positional patterns and pairwise effect sizes were illustrated in Figures 2, 3. Significant position effects were observed for TDC, TDC/min, HSR, HSR/min, HMLD, HMLD/min, MP/min, PL, and PL/min (*p* < 0.05). No significant position effects were found for MP, HIA, HIA/min, HID, or HID/min.

**Table 2:** Descriptive and inferential statistics for external load during the standardized pre-match warm-up according to playing position.

External load metric	Back	Pivot	Wing	Positon group effect		
				F/H	*p*-value	*η_p_*^2^/*ε*^2^
TDC (m)	1,137.8 ± 171.3	1,092.1 ± 157.8	1,199.6 ± 120.1	3.18	0.046	0.06
HSR (m)	10.6 ± 15.1	7.2 ± 21.5	19.4 ± 14.8	4.30	0.016	0.08
HMLD (m)	67.1 ± 33.1	47.0 ± 37.6	86.0 ± 32.1	8.43	<0.001	0.14
MP (W·kg^−1^)	3,049.7 ± 737.9	2,888.7 ± 423.3	3,186.3 ± 440.0	1.43	0.244	0.03
HIA (n)	3.5 ± 3.1	4.2 ± 6.2	4.0 ± 2.5	0.31	0.735	0.01
HID (n)	2.5 ± 2.4	3.1 ± 4.9	2.9 ± 2.0	0.44	0.647	0.01
PL (a.u.)	15.8 ± 2.8	17.1 ± 2.5	18.1 ± 3.3	6.31	0.003	0.11
TDC/min (m·min^−1^)	62.3 ± 6.8	55.8 ± 7.8	62.4 ± 6.5	7.29	0.001	0.12
HSR/min (m·min^−1^)	0.6 ± 0.8	0.4 ± 1.1	1.0 ± 0.7	4.17	0.018	0.07
HMLD/min (m·min^−1^)	3.6 ± 1.6	2.4 ± 2.0	4.5 ± 1.6	8.84	<0.001	0.15
MP/min (W·kg^−1^·min^−1^)	166.2 ± 31.3	147.6 ± 21.1	165.6 ± 21.4	3.60	0.031	0.07
HIA/min (n·min^−1^)	0.2 ± 0.2	0.2 ± 0.3	0.2 ± 0.1	0.27	0.761	0.01
HID/min (n·min^−1^)	0.1 ± 0.1	0.2 ± 0.2	0.1 ± 0.1	0.39	0.677	0.01
PL/min (a.u.·min^−1^)	0.9 ± 0.1	0.9 ± 0.1	0.9 ± 0.2	3.08	0.050	0.06

Values are mean ± standard deviation. *η_p_*², partial eta squared; *ε*^2^, epsilon-squared; TDC, total distance covered; HSR, high-speed running; HMLD, high metabolic load distance; MP, metabolic power; HIA, high-intensity accelerations; HID, high-intensity decelerations; PL, PlayerLoad; a.u., arbitrary units.

Overall, wings exhibited the highest external load values during the warm-up. They covered more TDC than pivots (*p* = 0.044, ES = 0.69), accumulated greater HSR and HMLD than backs (*p* = 0.044, ES = 0.54) and pivots (*p* = 0.027, ES = 0.75), and showed higher HSR/min than pivots (*p* = 0.027, ES = 0.75). Both wings and backs recorded greater TDC/min and HMLD/min than pivots (*p* = 0.001–0.003, ES = 0.94–0.96), while backs also showed higher MP/min than pivots (*p* = 0.027, ES = 0.75). In addition, wings registered higher PL and PL/min than backs (*p* = 0.002–0.045, ES = 0.54–0.78).

**Figure 4:**
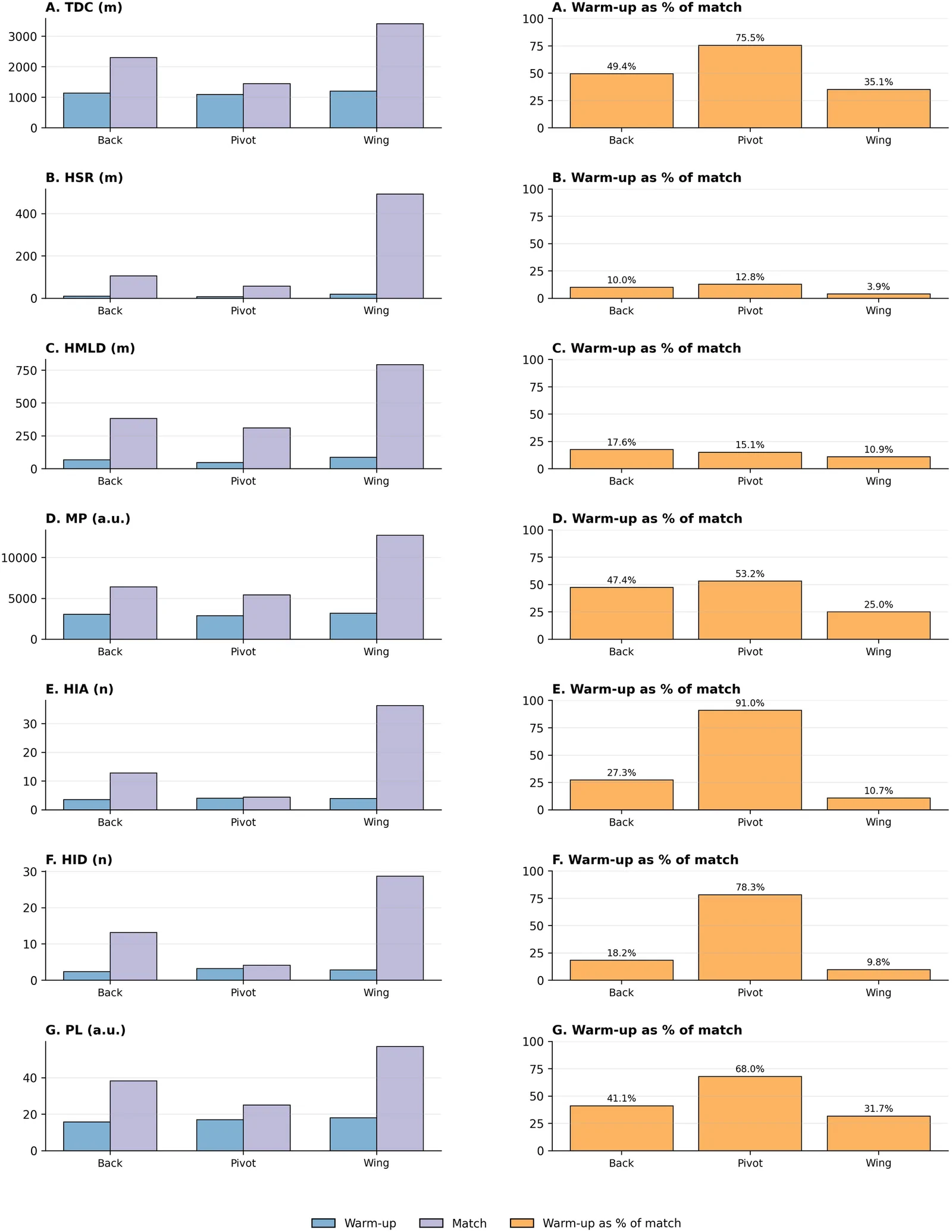
Comparison of absolute external load and relative warm-up contribution to match load by playing position.

The external load accumulated during the pre-match warm-up was also compared with that registered during match play according to playing position in Figure 4 Overall, the warm-up accounted for a considerable proportion of match load in several variables, although this contribution varied by playing positions. Pivots generally showed the highest relative contribution of warm-up to match load, whereas wings showed the lowest values in most metrics, particularly in HSR, HIA, and HID. In absolute terms, the difference between match and warm-up load was greater in wings than in backs and pivots across most variables.

## 4 Discussion

To the best of the authors’ knowledge, this is the first study to examine position-specific external-load demands during a standardized pre-match warm-up in women's handball. The aims of the present study were to compare external load during the warm-up among playing positions and to quantify the contribution of warm-up load relative to subsequent match demands. The main results revealed significant position-related differences in several external load variables, despite a similar warm-up duration across positions. Overall, wings showed the greatest external load, particularly in TDC and TDC/min, HSR, HMLD, and PL, whereas pivots consistently presented the lowest values. Backs also exhibited higher TDC/min, HMLD/min, and MP/min than pivots. In contrast, the relative contribution of warm-up to match load was generally greater in pivots and lower in wings, especially in TDC, MP, HIA, HID and PL. These findings suggest that the pre-match warm-up in women's handball is not uniformly distributed across playing positions and may reflect position-specific movement patterns and preparatory needs that should be considered in applied load monitoring. Additionally, these results indicate that warm-up load should not be interpreted merely as a preparatory routine, but also as a meaningful component of the total external load accumulated on match day.

One of the most notable findings is the significant influence of playing positions on external load during pre-match warm-up. Specifically, wings showed the greatest external load, while pivots consistently presented the lowest values. Backs appeared to occupy an intermediate profile, although they still showed greater relative distance-, metabolic-, and power-related values than pivots. It is difficult to compare the findings of the present study with previous research because there is a lack of scientific evidence comparing differences in external load pre-match warm-up between playing positions in handball. However, these positional profiles are consistent with external load registered during match play, where wings presented the highest external load, while pivots showed the lowest values (12, 13, 23, 24). These results are consistent with the recent volleyball literature (19), which also showed clear positional differences in pre-match warm-up load, with some roles accumulating greater locomotive and neuromuscular demands than others, reinforcing that a standardized warm-up protocol does not necessarily result in a similar external load across positions.

A plausible explanation for these positional differences observed during the warm-up could be attributed to the interaction of multiple factors. First, because warm-up duration did not differ between playing positions, the observed differences were likely related to the intensity and execution of the tasks rather than to unequal exposure time. Players may have spontaneously adjusted the intensity, velocity, or execution of the standardized exercises according to their habitual positional roles, suggesting a degree of self-regulation within the same protocol. Second, the spatial organization of the throwing drills may have contributed to the greater load observed in wings, who started from wider positions and may therefore have covered longer distances when retrieving balls and returning to their starting positions. Third, the use of an absolute running-speed threshold (>18.1 km·h^−1^) to quantify HSR distance may also have influenced the positional comparisons. Pivots are generally taller and heavier than wings and typically achieve lower maximal running speeds during competition. Recent evidence from elite handball competitions showed that pivots reached lower maximal velocities than wings and backs, reflecting meaningful position-specific differences in sprint capacity (14, 15). Consequently, the same absolute HSR threshold may represent a substantially greater proportion of maximal speed for pivots than for faster players. This could reduce the number of pivot actions classified as HSR, even when those actions represent a relatively high locomotor intensity for the individual player. This methodological issue may be particularly relevant because some of the largest positional differences observed in the present study were found for HSR and HMLD. Recent methodological literature has emphasized that absolute running-speed thresholds do not account for inter-individual differences in maximal speed and may therefore result in different interpretations of the external load experienced by players (29).

As previously mentioned, the number of studies examining pre-match warm-up load in handball remains scarce, however the available evidence from other team-sports (e.g., football and volleyball) and the present findings converge in showing that warm-up is not a trivial pre-competition phase (19, 20). Instead, it represents a measurable external load exposure with the potential to reproduce part of the physical demands later observed during match play. Indeed, recent evidence from elite women's volleyball demonstrated clear position-specific differences in a standardized pre-match warm-up load, supporting the idea that athletes in different roles do not experience the same preparatory demands (19). Collectively, the available evidence and the present findings suggest that warm-up load should be monitored as a meaningful component of match-day external load rather than being regarded as a simple preliminary routine with minimal physical impact. Thus, including warm-up external load in match-day monitoring may help practitioners quantify player load more accurately and better prescribe the subsequent training load across the microcycle, particularly in the first post-match session (MD + 1), in which training content is often adjusted according to the load accumulated during match-day (24). Also, as in other team sports (e.g., soccer and basketball) (30–32), this may be especially relevant when differentiating between starters and non-starters, as considering only match exposure could underestimate the total load experienced by players with limited playing time and compromise the appropriate prescription of recovery or compensatory training interventions during the initial phase of the microcycle.

An additional relevant finding is that the relative contribution of warm-up external load to match load differed markedly between positions. Pivots generally showed the highest relative contribution of warm-up load, whereas wings showed the lowest values across most variables. This should not be interpreted as indicating that pivots performed a more demanding warm-up in absolute terms, but rather that the load accumulated during the warm-up represented a larger proportion of their subsequent match demands. A possible explanation for this difference may be related to the combination of two factors (1): the positional nature of pivot play during the match, which is largely based on static or quasi-static actions performed in highly restricted spaces near the 6-m line with short displacements, such as blocking, screening, and gaining position against opponents with frequent and strenuous body contacts (15), and (2) the standardized pre-match warm-up applied in the present study, in which pivots were exposed to the same running- and movement-based tasks as the rest of the players, including high-intensity accelerations, decelerations, and sprint-related actions without the ball.

Additionally, in terms of volume metrics, pivots exhibited a particularly high relative contribution of warm-up load to match load in TDC and PL, reaching approximately 70%–75% of the corresponding match values. This may be practically relevant, as pivots are typically heavier and larger players, and the accumulation of a high locomotor and mechanical load during a short warm-up period may place substantial stress on the lower limbs (2). Conversely, wings showed the greatest absolute difference between warm-up and match load in most variables, indicating that their competitive demands increased much more markedly once the match started. A similar analytical perspective has recently been used in football (20) with similar findings, where pre-match warm-up load represented a variable proportion of match load depending on the selected metric, ranging from approximately 5% in high-speed variables to around 15%–20% in acceleration- and load-based variables. Taken together, these findings suggest that the same standardized warm-up may have a different practical meaning depending on the playing position. In other words, the warm-up should be interpreted not only according to its absolute magnitude, but also according to how much of the subsequent match load it already represents for each playing position. Therefore, as indicated by several researchers, it may be necessary to examine whether the neglected pre-match warm-up external load is an indicator of achieving optimal performance other than preparing the players for the match or whether it has a routine that may cause unnecessary fatigue (18, 19).

### 4.1 Strengths and limitations

The present study has several strengths. To the best of the authors’ knowledge, it is the first investigation to quantify position-specific external load during a standardized pre-match warm-up in women's handball. Data were collected during official competition across multiple matches, increasing the ecological validity of the findings. The same warm-up structure, sequence, and duration were maintained throughout the competitive period, with the protocol led by the team's strength and conditioning coach and supervised by a researcher. The start and end of each warm-up were identified using researcher-recorded timestamps, allowing the analyzed period to be clearly distinguished from any individual activities performed before or after the standardized protocol. In addition, the combination of LPS- and IMU-derived variables enabled both locomotor and accelerometer-based demands to be examined in absolute and relative terms.

Nevertheless, the present findings should be interpreted considering some limitations. First, the study included a relatively small sample (*n* = 22) from a single women's team competing in the Spanish 2nd National Division and only home matches were investigated. Therefore, the findings should be generalized to other teams, competitive levels, and match contexts with caution. Third, the LPS- and IMU-derived variables used in the present study did not comprehensively capture all the mechanical load of handball. Physical contacts, blocking, and screening may occur without substantial displacement or acceleration and may therefore be underestimated, particularly in pivots. Fourth, the study quantified only external load variables. Internal load during the standardized warm-up was not assessed through heart rate or perceptual measures. Consequently, no conclusions can be drawn regarding the overall effectiveness of the warm-up or its influence on physiological strain, psychological readiness, cognitive preparedness, perceived readiness to perform, or other non-physical components of competition preparation. Fifth, repeated observations from the same players and matches were analysed as individual records using one-way ANOVA or Kruskal–Wallis tests, consistent with previous pre-match warm-up studies (19, 20). Future research with larger samples should confirm these findings using hierarchical models that account for observations nested within players and matches.

### 4.2 Practical applications and future research

From a practical perspective, the present findings indicate that practitioners should not assume that a standardized pre-match warm-up elicits the same external load in all players. Accordingly, adapting pre-match warm-up routines by playing position may help optimize readiness while limiting unnecessary fatigue or overload. Consequently, physical trainers and coaches should design and implement a structuring position-based warm-up protocol into three different phases. First, a general phase should include mobility exercises, running drills, strength exercises (e.g., squats, lunges, bridges, planks and push-ups); knee stability tasks (e.g., single- and double-leg landing exercises) and change of direction drills to enhance neuromuscular readiness and reduce injury risks (18, 33). Second, a position-specific phase should reproduce the mainly movement patterns and technical activity profile of each position during match-play. For example, wings may benefit from a greater emphasis on long-distance running actions that elicit high-speed running (23), whereas backs may require more multidirectional actions, such as accelerations, decelerations, and changes of direction over very short distances and in tight spaces (23), to replicate the high mechanical and neuromuscular stress imposed to the lower-limbs. In contrast, pivots may benefit more from strength- and power-oriented exercises that reproduce high-intensity impacts and repeated physical body contacts, such as static blocking actions and maximal isometric muscle contractions, while reducing the proportion of running-based exercises over longer distances (6, 15). Finally, a third phase should integrate handball-specific ball exercises, such as passing drills, goalkeeper-specific warm-up, position-specific throws, and individual fast-breaks. In addition, strength and conditioning coaches and technical staff should consider not only the content of the warm-up, but also its duration, as previous research suggested that shorter protocols (∼15 min) may be more effective than longer ones (>20 min) for enhancing readiness without generating excessive pre-match fatigue (18, 34).

Future research should include larger samples, multiple teams, away matches, players from higher competitive levels, and goalkeepers to determine whether the observed positional patterns remain consistent across different competitive contexts and playing roles. Future studies should also combine external load with internal load data (e.g., heart-rate or perceptual measures) to provide a more comprehensive assessment of pre-match warm-up demands. In addition, experimental or crossover designs should compare standardized and individualized or position-specific warm-up strategies to determine whether modifying warm-up content improves competition readiness, subsequent match performance, and total match-day load management. Lastly, future studies in handball could complement absolute thresholds with normalized thresholds expressed as a percentage of each player's individual maximal speed. This reference could be obtained from the highest valid speed recorded across training and competition or from a standardized linear sprint test, such as a 20- or 30-m sprint. However, the method used to determine maximal speed should be clearly reported because peak match speed and sprint-test speed represent related but not identical reference values.

## 5 Conclusions

In conclusion, the present study shows that the external load registered during a standardized pre-match warm-up protocol is position-specific and should not be considered a uniform preparatory stimulus across players. While wings exhibit the greatest absolute warm-up demands, pivots accumulated the highest relative contribution of warm-up external load in relation to match demands. These findings indicate that warm-up load represents a meaningful component of total match-day external load and should be designed and structured according to the specific technical and physical match-demands of each playing position to optimize readiness, enhance performance and minimize fatigue.

## Data Availability

The data generated and analyzed during the present study are not publicly available due to ethical restrictions but are available from the corresponding author upon reasonable request. Requests to access the datasets should be directed to c.gsanchez@upm.es.
